# Micro-Expression Recognition via LoRA-Enhanced DinoV2 and Interactive Spatio-Temporal Modeling

**DOI:** 10.3390/s26020625

**Published:** 2026-01-16

**Authors:** Meng Wang, Xueping Tang, Bing Wang, Jing Ren

**Affiliations:** Faculty of Information Engineering and Automation, Kunming University of Science and Technology, 727 South Jingming Road, Kunming 650500, China; tangxueping@stu.kust.edu.cn (X.T.); 20232204231@stu.kust.edu.cn (B.W.); renjing@stu.kust.edu.cn (J.R.)

**Keywords:** micro-expression recognition, LoRA, spatiotemporal modeling, Fourier transform, graph attention network

## Abstract

Micro-expression recognition (MER) is challenged by a brief duration, low intensity, and heterogeneous spatial frequency patterns. This study introduces a novel MER architecture that reduces computational cost by fine-tuning a large feature extraction model with LoRA, while integrating frequency-domain transformation and graph-based temporal modeling to minimize preprocessing requirements. A Spatial Frequency Adaptive (SFA) module decomposes high- and low-frequency information with dynamic weighting to enhance sensitivity to subtle facial texture variations. A Dynamic Graph Attention Temporal (DGAT) network models video frames as a graph, combining Graph Attention Networks and LSTM with frequency-guided attention for temporal feature fusion. Experiments on the SAMM, CASME II, and SMIC datasets demonstrate superior performance over existing methods. On the SAMM 5-class setting, the proposed approach achieves an unweighted F1 score (UF1) of 81.16% and an unweighted average recall (UAR) of 85.37%, outperforming the next best method by 0.96% and 2.27%, respectively.

## 1. Introduction

Facial expressions serve as intuitive indicators of human emotions, typically triggered by specific stimuli and reflecting emotional changes spontaneously or involuntarily [1]. Unlike macro-expressions with longer durations, micro-expressions last less than 0.5 s, exhibit low intensity, and are difficult to detect, often linked to suppressed emotions [2]. Therefore, micro-expression recognition (MER) has a wide range of applications in affective computing, psychological analysis, security monitoring, and clinical diagnosis [3,4,5]. Specifically, as hard-to-fake cues of genuine emotions, micro-expressions can aid psychological health monitoring through early screening and non-invasive monitoring of depression, anxiety, and PTSD, as well as pathological examination through pain assessment and lie detection, thereby providing objective emotional indicators. However, their complex spatio-temporal characteristics, including uneven spatial frequency distributions (e.g., low-frequency contours vs. high-frequency textures) and non-uniform temporal dynamics, pose significant challenges in computer vision. This paper focuses on modeling subtle dynamic features and enhancing model robustness.

Early traditional MER methods relied primarily on hand-crafted feature extraction. Non-optical flow approaches, such as Local Binary Patterns on Three Orthogonal Planes (LBP-TOP) [6,7,8], described facial textures via pixel values for effective analysis; optical flow methods [9,10,11] extracted motion information from video sequences using deformation features. Although these methods achieved initial success, they suffered from complex feature design and limited robustness to noise, illumination variations, and individual differences. The advent of deep learning provided new momentum for MER, such as Liong et al.’s [12] Shallow Triple-Stream Three-Dimensional Convolutional Network (STSTNet), leveraging optical flow to achieve superior performance on CASME II, yet it depended on intricate preprocessing, resulting in high computational costs. Subsequently, Chen et al. [13] introduced the Block-Division Convolutional Network (BDCNN), reducing such dependency through implicit semantic augmentation, but overlooking global spatio-temporal coupling and thus constraining the modeling of non-uniform temporal dynamics. To further optimize computational complexity and accuracy, Xia et al. [14] developed the Recursive Convolutional Network (RCN), employing attention mechanisms to enhance parameter efficiency and cross-dataset robustness, though still hindered by class imbalance and small sample sizes.

Subsequently, self-supervised learning minimized labeled data needs; Wiles et al. [15] extracted attribute embeddings to establish a transfer foundation for downstream MER tasks, and Li et al. [16] detected facial action units from unlabeled videos. These early self-supervised paradigms marked a shift from supervised to unsupervised MER, yet their application to micro-expression-specific tasks requires further refinement. With the emergence of Vision Transformers (ViT) and pretrained models, Nguyen et al. [17] proposed Micron-BERT (μ-BERT), utilizing Transformer-based local micro-motion detection but neglecting spatial frequency variations. Similarly, Fan et al.’s [18] SelfME enhanced key region detection via symmetry, lacking decoupled temporal modeling. Peng et al. [19] and Verma et al. [20] improved generalization with transfer learning, yet stability under uncontrolled conditions remains insufficient. Although self-supervision and transfer learning diminish preprocessing dependencies and enhance generalization, existing methods remain unstable under uncontrolled conditions [21]. In general, key limitations include the following: models assume uniform spatial distribution, ignoring micro-expression frequency characteristics (e.g., low-frequency contours vs. high-frequency textures), hindering low-intensity motion capture; coupled spatio-temporal modeling lacks decoupled interaction optimization, impeding the accurate representation of non-uniform temporal dynamics, and small-sample and class imbalance issues heighten generalization risks in uncontrolled settings.

To address these issues, this paper proposes an innovative MER framework. It takes RGB video sequences as input, outputs micro-expression labels, and realizes efficient dynamic modeling and robust recognition via parallel extraction and interaction optimization of decoupled spatio-temporal features. Inspired by the strengths of self-supervised large models [22] and graph networks in capturing complex patterns, this framework efficiently resolves low-intensity feature extraction, temporal dependency modeling, and small-sample generalization challenges. Core innovations include the following:

1. A parameter-efficient transfer strategy is introduced by fine-tuning only low-rank matrices within the last attention layers of DinoV2, achieving effective representation learning for MER.
2. We design a learnable frequency–spatial fusion module that performs adaptive Fourier decomposition and expert-based weighting to dynamically emphasize subtle high- and low-frequency cues crucial for micro-expressions.
3. A frequency-guided temporal modeling module integrates graph attention and LSTM to capture temporal dependencies modulated by frequency responses, forming a closed-loop interaction between spatial, frequency, and temporal domains.

To further clarify the advancements of our approach over existing MER studies, a detailed conceptual comparison and theoretical analysis are provided in Appendix A.

## 2. Related Work

Micro-expression recognition (MER) aims to classify micro-expressions from video sequences. Early MER methods relied on hand-crafted feature extraction, characterizing micro-expressions through facial texture changes or inter-frame motion analysis. Local Binary Patterns on Three Orthogonal Planes (LBP-TOP) [6,7,8] extracted local texture features across three orthogonal planes, effectively describing dynamic facial textures and achieving initial success in MER. Optical flow-based methods [9,10,11] captured subtle facial variations using inter-frame motion information; for instance, Liong et al. [9] analyzed micro-expression dynamics with apex frame optical flow, enhancing recognition accuracy. Though effective in specific scenarios, these approaches depended on labor-intensive design and exhibited limited robustness to noise, illumination variations, and individual differences.

The advent of deep learning introduced novel MER techniques. Ref. [23] pioneered deep learning-based MER, employing CNN models for object- and expression-based transfer learning followed by evolutionary feature selection. Gan et al. [24] proposed OFF-ApexNet, a deep learning framework leveraging optical flow features between onset and apex frames to improve recognition performance. Liong et al. [12] developed a Shallow Triple-Stream Three-Dimensional CNN (STSTNet), utilizing optical flow and strain features as input streams to enhance dynamic facial action capture, thereby boosting accuracy. Zhou et al. [25] designed a Dual Inception Network for cross-database MER, processing horizontal and vertical optical flow features with two Inception net-works, demonstrating robustness across datasets. Recent research has shifted toward advanced deep learning techniques. Zhou et al. [26] introduced a feature refinement method with expression-shared and expression-specific modules to fuse MER features, achieving state-of-the-art results on benchmark datasets. Lei et al. [27] explored facial graph representation learning combined with action unit fusion, improving the model’s ability to capture subtle relationships between facial landmarks. Kumar and Bhanu [28] advanced this approach using a Graph Attention Convolutional Network to exploit connections between landmarks and optical flow patches, showing significant improvements on CASME II and SAMM datasets. Liu et al. [29] proposed a lightweight ViT model enhanced by transfer learning for MER, highlighting potential in cross-domain applications.

Recently, self-supervised learning, capable of learning robust feature representations without extensive labeled data, has opened new avenues in facial visual tasks. Li et al. [16] extracted facial action unit features from unlabeled videos using self-supervised representation learning, enhancing detection of subtle facial movements for MER-related tasks. Wiles et al. [15] developed a video-based self-supervised method for facial attribute embedding, capturing dynamic features via temporal modeling and offering transfer learning insights for MER. Building on these efforts, Verma et al. [20] designed LEARNet, a dynamic imaging network based on incremental learning, employing component-aware attention to focus on key facial regions, improving micro-expression feature expressiveness. Nguyen et al. [17] introduced Micron-BERT (μ-BERT), utilizing Diagonal Micro-Attention (DMA) and Patch of Interest (PoI) modules for self-supervised micro-movement detection, achieving superior performance across benchmarks. Fan et al. [18] proposed the SelfME framework, integrating self-supervised motion learning with a Symmetric Contrastive Visual Transformer (SCViT) to enhance facial symmetry representation, significantly improving MER performance.

Additionally, fine-tuning techniques based on large models have gained attention in recent MER research. DinoV2 [22], a self-supervised pretrained Vision Transformer (ViT), learns robust visual representations from large-scale unlabeled data, finding wide application in fine-grained image tasks. LoRA [30] optimizes pretrained models by introducing low-rank update matrices, reducing parameter adjustments while preserving generalization, making it suitable for resource-constrained fine-tuning scenarios. Recently, large-scale foundation models and vision–language models (VLMs) have shown strong generalization in medical and affective computing. Surveys such as Vision–Language Models in Medical Image Analysis [31] highlight the transition from multimodal fusion to general large models.

## 3. Methods

**Figure 1:**
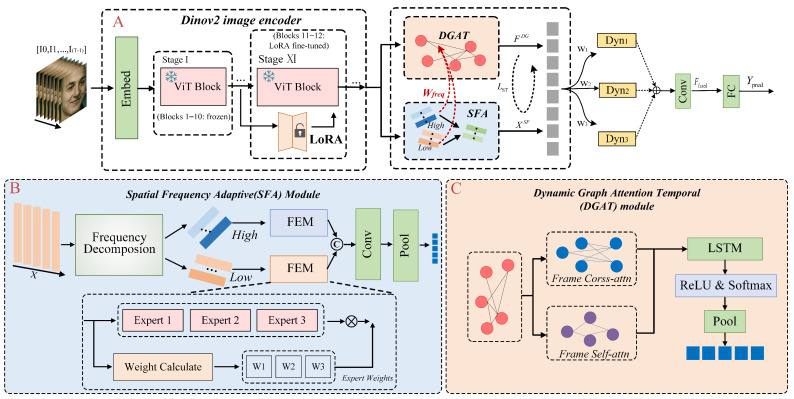
Overall framework of the proposed micro-expression recognition approach. (**A**) LoRA-enhanced DinoV2 encoder for frame-level feature extraction. (**B**) Spatial Frequency Adaptive (SFA) module that decomposes features into low- and high-frequency components and performs adaptive frequency fusion. (**C**) Dynamic Graph Attention Temporal (DGAT) network for frequency-guided temporal dependency modeling and aggregation.

This paper proposes a novel MER framework that efficiently models micro-expressions through parallel extraction and interactive optimization of decoupled spatio-temporal features. The overall architecture is illustrated in Figure 1. Given an input video sequence ${\left\{I_{t}\right\}}_{t=1}^{T}$, each frame $I_{t}$ is processed by a LoRA-tuned DinoV2 to extract frame-level embeddings, forming the feature sequence $X=\left[f_{1},f_{2},\ldots ,f_{T}\right]\in R^{T\times D}$. The sequence *X* is then fed into two parallel branches: **SFA** performs a 2D fast Fourier transform to decompose *X* into low- and high-frequency components, which are adaptively fused via an expert-gating mechanism to produce the spatially enhanced feature $X^{SF}\in R^{T\times D}$ and the frequency weight matrix $W_{freq}\in R^{T\times T}$. **DGAT** constructs a temporal graph with frames as nodes and modulates edge weights using $W_{freq}$ to model frequency-guided temporal dependencies, generating the temporal representation $F^{DG}\in R^{T\times D}$. Finally, $X^{SF}$ and $F^{DG}$ are fused into a unified representation for classification.

Figure 1 illustrates the MER framework with decoupled spatio-temporal modeling and optimization, addressing low-intensity motion capture, as detailed in Section 3.1, Section 3.2, Section 3.3 and Section 3.4.

### 3.1. DinoV 2 Image Encoder Feature Extraction

Self-supervised Vision Transformers like DinoV2 provide robust visual representations, but fine-tuning on small MER datasets is computationally expensive. LoRA enables parameter-efficient adaptation, motivating its integration in our framework.

For the MER task, RGB video sequences serve as input. To match DinoV2 encoder requirements, all frames are extracted from each video, yielding a chronological sequence $\left\{I_{0},I_{1},\ldots ,I\left(N-1\right)\right\}$, where *N* is the total frame count. These are uniformly sampled to T=8 frames with interval [N/T] to reduce computational complexity, resulting in $\left\{I_{0},I_{1},\ldots ,I\left(T-1\right)\right\}$. Uniform temporal sampling with a fixed length T=8 provides a compact and stable input representation. Although it may reduce temporal coverage for very long videos, micro-expression clips are typically short, and uniform sampling is widely adopted in prior MER studies.

To address the challenges of subtle expressions and brief durations in MER, we employ a self-supervised pretrained Vision Transformer (ViT), DinoV2 [22], optimized via Low-Rank Adaptation (LoRA) [30]. This preserves DinoV2’s generalization while infusing task-specific knowledge for fine-grained spatio-temporal feature capture. The encoder structure is shown in Figure 1A.

DinoV2 comprises L=12 Transformer blocks, each with multi-head attention, a feedforward network, and layer normalization. The first ten blocks remain frozen, while the final two undergo LoRA fine-tuning to align with micro-expression tasks, as higher layers yield more task-relevant features. For input features $X^{\left(l\right)}\in R^{B\times C\times H\times W}$ at layer *l*, the output after the Transformer blocks is

(1) $$X_{\mathrm{final}}=f_{\mathrm{LN}}\;\left(X^{\left(L\right)}\right).$$

where $X^{\left(L\right)}\in R^{B\times C\times H\times W}$ denotes the output of the *L*-th block, *B* is the batch size, *C* the channel dimension, H×W is the spatial resolution, and $f_{\mathrm{LN}}$ is the layer normalization. For the final two layers, we fine-tune the Query (Q) and Value (V) matrices using LoRA, as this balances performance and efficiency [30]. The original weights $W_{Q}$ and $W_{V}$ remain frozen, while LoRA introduces low-rank update matrices $W_{Qd}$, $W_{Qe}$, and $W_{Vd}$, $W_{Ve}$, where $W_{Qd},W_{Vd}\in R$, with rank r=8. The fine-tuned *Q* and *V* are computed as

(2) $$\left\{ \begin{array}{c} Q=X^{\left(l\right)}\left(W_{Q}+W_{Qd}W_{Qe}\right)\\ V=X^{\left(l\right)}\left(W_{V}+W_{Vd}W_{Ve}\right) \end{array}\right.$$

Here, *K* retains the original weight $W_{K}$. During fine-tuning, only the low-rank matrices are optimized, minimizing parameters while adapting to micro-expression features. This yields deep features $f_{t}\in R^{D}$ per frame $I_{t}$, preserving generalization, reducing complexity, and providing high-quality spatial inputs for subsequent SFA and DGAT modules.

### 3.2. Spatial Frequency Adaptation Network (SFA)

In MER, capturing subtle facial texture variations is hindered by uneven spatial frequency distributions, where low-frequency components capture contours and high-frequency ones reveal local details. To address this, we introduce the SFA module, which enhances DinoV2 extracted features via Fourier-domain decomposition and expert networks, improving sensitivity to low-intensity motions.

SFA takes as input the global frame features $f_{t}\in R^{B\times D}$ extracted by DinoV2 and produces enhanced spatial features $X^{SF}\in R^{B\times T\times D}$, with dimensions determined by subsequent convolutional operations. The structure is illustrated in Figure 1B, and the process proceeds as follows: All frame-level features are stacked in temporal order to form a two-dimensional feature representation $X=[f_{0},f_{1},\ldots ,f_{T-1}]$. Subsequently, two-dimensional Fourier transform is applied to the feature representation *X* along the feature dimension *D* and the temporal dimension *T*, yielding the frequency-domain representation *F*, with the FFT transformation defined as

(3) $$F_{b,c}\left(u,v\right)=\sum_{d=0}^{D-1}\sum_{t=0}^{T-1}X_{b,c}\left(d,t\right)\;e^{-j2\pi \left(\frac{ud}{D}+\frac{vt}{T}\right)}$$

where u=0,…,D−1, v=0,…,T−1, *b*, and *c* denote batch and feature-channel indices, respectively, and $j=\sqrt{-1}$. Here, *D* denotes the embedding dimension of the frame-level feature $f_{t}$ produced by DinoV2, rather than a flattened spatial dimension. To separate low- and high-frequency components, we define a frequency mask $M\in R^{D\times T}$, where the frequency mask *M* is a fixed binary mask. Taking the center of the frequency domain as reference, a fixed cutoff radius *r* is defined, and all frequency components whose radial distance to the center does not exceed *r* are regarded as low-frequency regions and assigned a value of 1, while the remaining frequency components are assigned a value of 0. This mask is used to distinguish low- and high-frequency components.

For effective separation, we apply a center shift to *F*, relocating low-frequency components to the center, followed by frequency component isolation:

(4) $$\left\{\begin{array}{c} F_{\mathrm{high}}=F⊙\left(1-M\right)\\ F_{\mathrm{low}}=F⊙M \end{array}\right.$$

where ⊙ indicates element-wise multiplication. The representations are then reverted to the time domain via inverse shift and inverse FFT:

(5) $$\left\{ \begin{array}{cc} X_{\mathrm{high}}\left(b,c,d,t\right) & =\sum_{u=0}^{D-1}\sum_{v=0}^{T-1}F_{\mathrm{high}}\left(b,c,u,v\right)\;e^{j2\pi \left(\frac{ud}{D}+\frac{vt}{T}\right)}\\ X_{\mathrm{low}}\left(b,c,d,t\right) & =\sum_{u=0}^{D-1}\sum_{v=0}^{T-1}F_{\mathrm{low}}\left(b,c,u,v\right)\;e^{j2\pi \left(\frac{ud}{D}+\frac{vt}{T}\right)} \end{array}\right.$$

where $X_{\mathrm{low}},X_{\mathrm{high}}\in R^{B\times D\times T}$, and the real part is taken to obtain real-valued features. The calculation of frequency-domain weight is based on the energy distribution of high- and low-frequency components and is defined as

(6) $$W_{\mathrm{freq}}=softmax\left(\frac{∥X_{\mathrm{high}}{∥}_{2}^{2}+{∥X_{\mathrm{low}}∥}_{2}^{2}}{\max\left(∥X_{\mathrm{high}}{∥}_{2}^{2},{∥X_{\mathrm{low}}∥}_{2}^{2}\right)+∥X_{\mathrm{high}}{∥}_{2}^{2}+{∥X_{\mathrm{low}}∥}_{2}^{2}}\right)$$

A Frequency Enhancement Module (FEM) normalizes and refines components:

(7) $$X_{\mathrm{low}}^{\mathrm{norm}}=\frac{(X_{\mathrm{low}}-\mu )\cdot \gamma +\beta }{\sqrt{\sigma^{2}+\varepsilon }}$$

μ and $\sigma^{2}$ represent the mean and variance of the features, respectively, with $\varepsilon =10^{-6}$, and γ and β as learnable parameters. Normalized features undergo 2D convolution, splitting into components $X_{S}$ and $K_{S}$; subsequently, a mixture-of-experts-based modeling mechanism is introduced to adaptively model the frequency features, and the specific computation process is described as follows:

(8) $$\left\{ \begin{array}{cc} X_{\mathrm{expert}} & ={\mathrm{Conv}}_{1}\left(X_{s}\right)\\ K_{\mathrm{expert}} & ={\mathrm{Conv}}_{2}\left(K_{s}\right)\\ h_{i} & ={\mathrm{Conv}}_{3}\left(X_{\mathrm{expert}}⊙K_{\mathrm{expert}}\right) \end{array}\right.$$

$h_{i}\in R$ denotes the scalar response of the *i*-th expert, obtained by applying a channel-wise convolution followed by global aggregation. Each expert is implemented as lightweight convolutional layers designed to model frequency features from different parameterized perspectives. Specifically, ${\mathrm{Conv}}_{1}$ and ${\mathrm{Conv}}_{2}$ are applied to $X_{S}$ and $K_{S}$, respectively, and the interacted features are mapped to $h_{i}$, which reflects the activation strength of the corresponding expert for the current input sample. The same feature normalization and modeling process is applied to both low- and high-frequency components, differing only in their respective frequency inputs.

The gating network computes a scalar importance score for each expert by aggregating the gated responses over the temporal and feature dimensions, followed by softmax normalization across experts:

(9) $$\tilde{w}\left(b,n\right)=\frac{1}{DT}\sum_{d=1}^{D}\sum_{t=1}^{T}\left(W_{\mathrm{gate}}^{\left(n\right)}X_{S}\left(b,d,t\right)\right),$$

(10) $$w\left(b,n\right)={\mathrm{Softmax}}_{n}\left(\tilde{w}\left(b,n\right)\right),$$

where $W_{\mathrm{gate}}^{\left(n\right)}\in R^{1\times 1}$ denotes the gating parameter of the *n*-th expert, and softmax ensures $\sum_{n=1}^{N}w\left(b,n\right)=1$. These weights indicate the relative contribution of each expert to the final feature representation for a given input sample, enabling the model to adaptively fuse expert outputs according to the frequency characteristics of the input. Using N=3 experts achieves a balance between representation capacity and model complexity, providing complementary frequency modeling while mitigating overfitting on small-scale micro-expression datasets. The weighted expert aggregation refines frequency representations, producing final outputs $X^{SF}$.

Compared with fixed band-pass filters, the proposed design explicitly decomposes features into the frequency domain via FFT and enables sample-adaptive frequency modeling through a mixture-of-experts and gating mechanism, allowing the model to dynamically balance low- and high-frequency information according to the frequency distribution of different micro-expression sequences. Compared with directly learning frequency-domain convolutions, this design preserves frequency interpretability while reducing the risk of overfitting when learning complex filters on small-scale micro-expression datasets, and the multi-expert structure further enhances the stability and generalization of frequency representations through complementary modeling.

For clarity, the intermediate tensor shapes in the SFA module are summarized as follows: $f_{t}\in R^{B\times D}$ denotes frame-level features; stacking all frames yields $X\in R^{B\times D\times T}$; the low- and high-frequency components $X_{\mathrm{low}}$ and $X_{\mathrm{high}}$, as well as the frequency-enhanced features $X_{S}$, $K_{S}$, $X_{\mathrm{expert}}$, and $K_{\mathrm{expert}}$, all preserve the same temporal-feature structure; $h_{i}\in R$ denotes a scalar expert response; and $X^{SF}\in R^{B\times T\times D}$ is the final frequency-enhanced output.

### 3.3. Dynamic Graph Attention Temporal Network (DGAT)

In order to solve the spatio-temporal feature coupling, we propose an innovative DGAT module. This module integrates Graph Attention Networks [32] and LSTM, leveraging SFA feedback for interactive optimization to effectively capture inter-frame dependencies.

In this paper, DGAT takes as input the per-frame features $f_{t}$ extracted by DinoV2, producing enhanced temporal features $F^{DG}\in R^{B\times D}$, with dimensions determined by subsequent pooling operations. The DGAT module structure is depicted in Figure 1C.

To model temporal dependencies between frames, the *T* frames in a video sequence are treated as nodes in a graph, with feature dimension *D* serving as the node feature dimension. Based on this, a temporal-aware densely connected graph is constructed, whose adjacency matrix $A\in R^{T\times T}$ describes potential structural connections between frames, where $A_{ij}$ indicates the existence of a possible connection between the *i*-th and *j*-th frames. It should be noted that the adjacency matrix is only used to define the graph topology, while the actual relational strengths between nodes are adaptively learned through the subsequent multi-head graph attention mechanism.

The self-attention mechanism computes attention weights between each frame and its neighbors. For layer *l* of GAT, the feature update for frame *i* is

(11) $$F_{i}^{\left(l\right)}=\mathrm{ELU}\left(\sum_{j\in N(i)}\alpha_{ij}^{\left(l\right)}W^{\left(l\right)}F_{j}^{\left(l-1\right)}\right)$$

where N(i) denotes the neighbor set of node *i* (including itself), $W^{\left(l\right)}\in R^{D^{\prime }\times D}$ represents the linear projection matrix, and $D^{\prime }$ denotes the intermediate feature dimension, which is set to D/H for each attention head to maintain a balanced model capacity under multi-head attention. The attention coefficient $a_{ij}^{\left(l\right)}$ computes as

(12) $$\alpha_{ij}^{\left(l\right)}=Softmax_{j}\left(LeakyReLU\times \left(\alpha^{\left(l\right)}\cdot \left[W^{\left(l\right)}F_{i}^{\left(l-1\right)}∥W^{\left(l\right)}F_{j}^{\left(l-1\right)}\right]\right)\right)$$

where $a^{\left(l\right)}$ represents attention parameters, and ∥ indicates vector concatenation. DGAT employs a multi-head attention mechanism with *H* attention heads, concatenating outputs to the specified dimension.

To incorporate the frequency guidance from the SFA module into DGAT, the global frequency weight $W_{\mathrm{freq}}$ computed in SFA is applied at the frame level, where the weight of the *i*-th frame is denoted as $W_{\mathrm{freq}}\left(i\right)$. Based on this, a frame-wise frequency modulation term is constructed as

(13) $$W_{\mathrm{freq}}\left(i,j\right)=W_{\mathrm{freq}}\left(i\right)\cdot W_{\mathrm{freq}}\left(j\right),$$

which is used to measure the consistency of frequency responses between two frames. The resulting frequency modulation term is then injected into the attention computation. The updated attention score formula is

(14) $$\alpha_{ij}=Softmax\left(\frac{\left(h_{i}W_{Q}\right){\left(h_{j}W_{K}\right)}^{T}}{\sqrt{d_{k}}}+\eta W_{\mathrm{freq}}\left(i,j\right)\right)$$

where $\alpha_{ij}$ denotes the attention weight between the *i*-th and *j*-th frames, η∈R denotes a learnable scalar, $W_{\mathrm{freq}}\left(i\right)$ is computed from the high- and low-frequency distributions in the SFA module, and the pairwise term $W_{\mathrm{freq}}\left(i,j\right)$ is constructed accordingly; $h_{i},h_{j}\in R^{D}$ represent node features, $W_{Q},W_{K}\in R^{D\times d_{k}}$ are projection matrices, and $d_{k}$ indicates the attention dimension.

The cross-attention mechanism further aggregates multi-head outputs, yielding the final frame features:

(15) $$F_{i}^{\left(l\right)}=\mathrm{ELU}\left(\sum_{j\in N(i)}\beta_{ij}W_{\mathrm{out}}F_{j}^{\left(l-1\right)}\right)$$

Here, $F_{j}\in R^{D}$ represents the concatenated multi-head features. $W_{\mathrm{out}}\in R^{D\times (D\cdot H)}$ restores the output dimension. The attention coefficient $\beta_{ij}$ computes similarly. GAT output yields $F_{i}^{\left(l\right)}\in R^{B\times T\times D}$.

Specifically, DGAT first applies graph attention to model inter-frame relations, followed by temporal sequence modeling using an LSTM. We then feed the GAT output $F_{i}^{\left(l\right)}$ into the LSTM and obtain the final representation as

(16) $$F^{DG}=MaxPool\left(Softmax\;LSTM(F_{i}^{\left(l\right)})\right)$$

Finally, temporal max-pooling aggregates frame-level LSTM outputs into the video-level feature $F^{DG}$. We further evaluate the contribution of the LSTM by removing it and directly pooling the GAT outputs; detailed results are provided in Appendix A (Table A2).

This section achieves the precise capture of temporal dynamics through DGAT, serving as the framework’s temporal core. It interacts with upstream SFA spatial frequency information for fusion, ensuring coherence in downstream feature integration.

### 3.4. Feature Fusion and Multi-Task Loss Function

**Figure 2:**
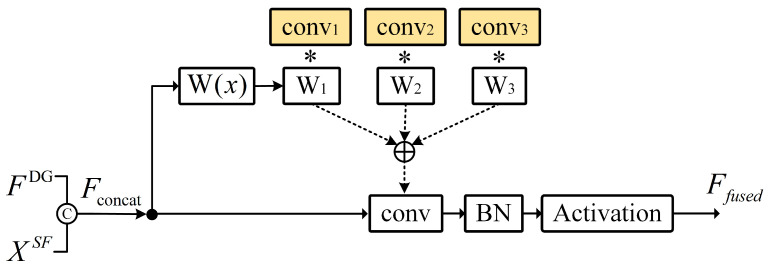
Detailed explanation of dynamic convolution. The symbol ∗ represents the operation between each convolutional output and its corresponding dynamic weight.

Figure 2 illustrates the dynamic convolution-based fusion mechanism used to integrate spatial frequency features and temporal representations.

To achieve effective synergy between the SFA and DGAT, we design an interactive fusion strategy based on dynamic convolution [33]. This approach integrates spatial features $X^{SF}$ and temporal features $F^{DG}$ into a unified representation. $X^{SF}$ and $F^{DG}$ concatenate along the channel dimension, generating a fused input:

(17) $$F_{\mathrm{concat}}=Concat\left(X^{SF},F^{DG}\right)$$

The concatenated features combine high- and low-frequency information with non-uniform inter-frame dynamics, providing rich information for dynamic convolution.

Dynamic convolution adaptively adjusts convolutional weights through an input-dependent kernel generator. A learnable fully connected layer $W_{k}\in R$ (where K=3 denotes the number of convolutional kernels) performs a linear transformation on $F_{concat}$, generating dynamic weights:

(18) $$K_{\mathrm{dyn},k}=Softmax\left(W_{k}\cdot F_{\mathrm{concat}}\right)$$

where $K_{\mathrm{dyn},k}$ represents the weight of the k−th convolutional kernel, dynamically dependent on the spatial and temporal distribution of $F_{\mathrm{concat}}$.

The dynamic convolution fusion formula is:

(19) $$F_{\mathrm{fused}}=\sum_{k=1}^{K}K_{\mathrm{dyn},k}⊙Conv_{k}\left(F_{\mathrm{concat}}\right)$$

Here, ⊙ denotes element-wise multiplication, and $Conv_{k}$ performs channel projection and feature reconstruction on $F_{\mathrm{concat}}$. The output $F_{fused}$ retains $R^{256}$ dimensional channels, compressing redundant information while preserving key spatio-temporal features. A subsequent fully connected layer projects to the number of classes *C*, generating predicted probabilities:

(20) $$Y_{\mathrm{pred}}=Softmax\left(FC(F_{\mathrm{fused}})\right)$$

The cross-entropy loss is defined as

(21) $$L_{CE}=-\frac{1}{B}\sum_{b=1}^{B}\log\left(Softmax{\left(Y_{\mathrm{pred}}\right)}_{b,Y_{\mathrm{true},b}}\right)$$

where $Y_{\mathrm{true}}\in R^{B}$ represents the ground truth labels.

To mitigate class imbalance and enhance feature distribution consistency, KL divergence regularization applies

(22) $$\left\{ \begin{array}{cc} L_{KL} & =-\frac{1}{B}\sum_{i=1}^{B}\log\left(d_{i}+\varepsilon \right)\\ d_{i} & =\min_{j\neq i}{∥F_{\mathrm{norm},i}-F_{\mathrm{norm},j}∥}_{2} \end{array}\right.$$

where $F_{\mathrm{norm}}$ denotes L2-normalized $F_{\mathrm{fused}}$, and $\varepsilon =10^{-6}$ serves as a smoothing term.

To strengthen synergy between SFA and DGAT, we introduce a spatio-temporal consistency loss, promoting semantic alignment between spatial and temporal features to enhance the modeling of micro-expression spatio-temporal dynamics. The spatio-temporal consistency loss is defined as

(23) $$L_{ST}=\frac{1}{B}\sum_{b=1}^{B}{∥X_{b}^{SF}-F_{b}^{DG}∥}_{2}^{2}$$

The final loss function combines classification loss, regularization loss, and spatio-temporal consistency loss with weighted contributions:

(24) $$L=L_{CE}+\lambda_{1}L_{KL}+\lambda_{2}L_{ST}$$

where $\lambda_{1}$ and $\lambda_{2}$ are hyperparameters balancing each loss term. Joint optimization enables the model to classify micro-expressions accurately while learning more robust and consistent spatio-temporal features.

## 4. Experimental Results and Discussion

### 4.1. Datasets

This study evaluates the proposed framework on three widely used benchmark datasets for micro-expression recognition: SAMM [34], CASME II [35], and SMIC [36]. Following common practice in the MER literature, the original emotion labels of SAMM and CASME II are reorganized into either three-class or five-class settings, while SMIC is evaluated under its original three-class setting. All samples are retained during re-labeling, and only the label space is modified. The exact number of samples per class after re-labeling is summarized in Table 1.

All experiments are conducted under the subject-independent Leave-One-Subject-Out (LOSO) evaluation protocol, which is the standard setting widely adopted in micro-expression recognition. Specifically, in each fold, all samples from one subject are used for testing, while samples from the remaining subjects are used for training.

### 4.2. Implementation Details and Evaluation Metrics

All experiments are implemented using the PyTorch (v1.13.1) framework and conducted on servers equipped with NVIDIA RTX 4090 GPUs. The model is optimized using the Adam optimizer with an initial learning rate of $1\times 10^{-4}$ and a batch size of 16. The DinoV2 encoder is initialized with pretrained weights and kept frozen, while LoRA modules with rank r=8 are fine-tuned. The remaining network components are trained in an end-to-end manner. Training is performed for up to 50 epochs with an early stopping strategy (patience = 10). The loss weighting parameters $\lambda_{1}=0.5$ and $\lambda_{2}=0.3$ are selected based on validation performance.

For data preprocessing, all frames are first extracted from each video and ordered temporally. A uniform temporal sampling strategy is then applied to select a fixed number of T=8 frames as input. Face detection and alignment are performed on each sampled frame using a pretrained 68-point facial landmark detector based on the Dlib library. The facial pose is normalized via an affine transformation computed from the eye centers. The aligned facial region is subsequently cropped, resized to a fixed spatial resolution, and normalized at the pixel level.

Given the limited scale of micro-expression datasets, lightweight data augmentation is applied only during training, including random horizontal flipping and mild affine transformations. No data augmentation is used for validation or testing.

To prevent test-set leakage during hyperparameter tuning, a subject-level validation strategy is adopted under the LOSO protocol. In each fold, the validation set is constructed exclusively from the training subjects, and all hyperparameters are selected based solely on validation performance. The held-out test subject is not involved in training or parameter tuning at any stage.

To comprehensively evaluate model performance in micro-expression recognition, we adopt two metrics: unweighted F1 score (UF1) and unweighted average recall (UAR). These metrics effectively assess classification capability and address class imbalance in micro-expressions. The formulas are

(25) $$\left\{ \begin{array}{cc} {\mathrm{Precision}}_{c} & =\frac{TP_{c}}{TP_{c}+FP_{c}}\\ {\mathrm{Recall}}_{c} & =\frac{TP_{c}}{TP_{c}+FN_{c}} \end{array}\right.$$

(26) $${F1}_{c}=2\cdot \frac{{\mathrm{Precision}}_{c}\cdot {\mathrm{Recall}}_{c}}{{\mathrm{Precision}}_{c}+{\mathrm{Recall}}_{c}}$$

(27) $$\mathrm{UF}1=\frac{1}{C}\sum_{c=1}^{C}F1_{c}$$

(28) $$\mathrm{UAR}=\frac{1}{C}\sum_{c=1}^{C}{\mathrm{Recall}}_{c}$$

where $TP_{c}$ denotes the number of correctly predicted samples for class *c*, $FP_{c}$ denotes the number of samples incorrectly predicted as class *c*, $FN_{c}$ denotes the number of class *c* samples misclassified as other categories, and *C* denotes the total number of classes.

### 4.3. Comparative Experimental Results

This section presents a detailed report of the experimental results for our proposed MRE framework on the SAMM, CASME II, and SMIC datasets. We systematically evaluate model effectiveness through performance comparisons, cross-dataset experiments, ablation studies, and parameter sensitivity analysis.

To comprehensively assess our method’s performance, we conduct comparative experiments against existing approaches for three-class and five-class expression recognition tasks. Table 2 summarizes results on the SAMM dataset, covering UAR and UF1 metrics.

Table 2 displays performance comparisons across methods on the SAMM dataset. Results indicate that our method achieves top performance in both tasks, with UF1 and UAR reaching 81.16% and 85.37% for five-class tasks, respectively, surpassing MiMaNet [40] (UF1 76.60%, UAR 78.49%) by 4.5% and 6.88%. While MiMaNet enhances spatio-temporal modeling with attention mechanisms, its adaptability to multi-category small-sample data is limited; our SFA module addresses this by decomposing frequency components and applying dynamic weighting to capture low-intensity facial motions, overcoming uneven spatial frequency distribution limitations. SAM-STN [38] (UF1 71.00%, UAR 77.33%) relies on fixed structures and optical flow preprocessing, struggling with complex dynamics, whereas our DGAT network improves robustness to non-uniform temporal dynamics via decoupled spatio-temporal modeling and frequency feedback optimization. Traditional methods like AMAN [37] (UF1 68.10%, UAR 69.83%) depend on hand-crafted features, struggling with diverse emotional variations and lagging significantly in accuracy.

For three-class tasks, our method attains UF1 of 82.93% and UAR of 83.77%, outperforming STANet [12] (UF1 77.25%, UAR 78.93%) by approximately 5.68% and 4.84%. These results validate the model’s capability to capture low-intensity and non-uniform dynamics of micro-expressions, demonstrating the framework’s superiority and robustness on the SAMM dataset.

Comparative experiments on the CASME II dataset demonstrate our method’s superior performance in both three-class and five-class tasks. Table 3 summarizes performance across methods for different task categories. For five-class tasks, our method achieves UF1 and UAR of 83.86% and 84.77%, respectively, outperforming the second-best method TSCNN (UF1 81.96%, UAR 82.20%) by 1.9% and 2.57%. TSCNN’s [43] multimodal fusion struggles with complex spatio-temporal dynamics, addressed by our SFA module’s frequency decomposition and dynamic weighting for low-intensity texture capture. SAM-STN [37] (UF1 79.63%, UAR 83.07%) and MiMaNet [40] (UF1 76.83%, UAR 79.38%) rely on fixed structures, faltering with non-uniform temporal dependencies, overcome by our decoupled spatio-temporal modeling and frequency feedback optimization.

For three-class tasks, UF1 and UAR reach 84.79% and 86.87%, respectively, surpassing MAE [41] (UF1 82.94%, UAR 84.08%) by 1.85% and 2.79%. MAE employs masked autoencoders for self-supervised learning, enhancing feature extraction, but its temporal modeling remains simplistic, inadequately addressing class imbalance. OFF-ApexNet [24] (UF1 82.87%, UAR 85.43%) and STSNet [12] (UF1 80.45%, UAR 78.68%) rely on optical flow features, incurring high computational costs and lacking robustness to illumination variations and individual differences. These results highlight that inadequate spatial frequency extraction and non-interactive temporal modeling limit other methods, affirming our framework’s superiority through decoupled feature extraction and interactive optimization.

Experiments on the SMIC dataset for three-class tasks similarly showcase our method’s excellence. Table 4 summarizes performance across methods on SMIC. Our method achieves UF1 and UAR of 85.72% and 84.17%, respectively, outperforming the second-best MAE (UF1 82.33%, UAR 81.09%) by 3.39% and 3.08%. Early methods DIKD [47] and TSCNN [43] struggle with dynamic capture due to convolutional limitations. Our SFA module enhances spatial features via frequency decomposition, while the DGAT network optimizes dynamics through decoupled spatio-temporal modeling. MTMNet [39] and AMAN [37] improve through temporal enhancement but remain constrained by spatial feature extraction. MiMaNet [40] and DSTAN [45] introduce attention mechanisms, gradually boosting performance (UF1 78.26% and 78.70%), yet underperform compared to our method in rapid dynamics and minority class recognition. MAE [41], as a self-supervised approach, nears our model’s performance (UF1 82.33%, UAR 81.09%), but its temporal modeling and class optimization fall short of optimality. Results align with CASME II (Table 2) and SAMM (Table 1), confirming our innovations’ superiority.

### 4.4. Cross-Dataset Generalization Experiments

Existing micro-expression methods progress on single datasets but lack cross-dataset generalization due to feature shifts and temporal mismatches. We conduct cross-dataset experiments to test our framework’s robustness, assessing adaptability to target datasets post-source training and quantifying innovative mechanism contributions.

Experiments adopt a transfer learning paradigm: the model trains on the source dataset (SAMM) and subsequently tests directly on target datasets (CASME II and SMIC). We incorporate a simple domain adaptation strategy based on consistency loss, integrating unlabeled samples from the target dataset into loss computation during training. The extended consistency loss $L_{\mathrm{consistency}}^{\mathrm{da}}$ aligns source–target feature distributions:

(29) $$L_{\mathrm{consistency}}^{da}=\frac{1}{B_{s}+B_{t}}\sum_{b=1}^{B_{s}+B_{t}}\left(∥X_{b}^{SF,s}-X_{b}^{SF,t}{∥}_{2}^{2}+∥F_{b}^{DG,s}-F_{b}^{DG,t}{∥}_{2}^{2}\right)$$

where $B_{s}$ and $B_{t}$ denote source and target batch sizes, respectively, and $X_{b}^{SF,s}$ and $F_{b}^{DG,s}$ represent the outputs of the SFA and DGAT of the b-th sample in the source domain. SFA frequency-domain weights $W_{\mathrm{freq}}$ feedback to DGAT, guiding domain alignment.

Table 5 compares our method with the baseline GRAPH-AU [X], a graph attention-based MER approach. For SAMM training and CASME II three-class testing, our method achieves UF1 and UAR of 80.15% and 78.88% without domain adaptation, improving to 82.67% and 83.45% with adaptation; GRAPH-AU scores 74.67% and 76.03%. Despite modeling inter-frame relationships with graph structures, GRAPH-AU lacks frequency priors and dynamic optimization, reducing accuracy under cross-domain shifts. For SAMM training and SMIC three-class testing, our method yields UF1 and UAR of 82.03% and 82.67% without adaptation, rising to 84.56% and 83.78% with adaptation; GRAPH-AU reaches 79.75% and 80.34%, lagging by 3.58% and 2.33%. GRAPH-AU’s weak temporal modeling on high-frame-rate data (e.g., SMIC) limits generalization. Our framework mitigates domain shifts via SFA frequency adaptation and DGAT dynamic optimization, with domain adaptation enhancing spatio-temporal consistency, demonstrating its potential to address generalization challenges.

### 4.5. Ablation Experiments

To validate the contributions of individual components in the MRE framework, we conduct ablation experiments on the CASME II three-class task, assessing the roles of LoRA, SFA, and DGAT. Table 6 lists UF1 and UAR results under different configurations.

Table 6 reveals that the baseline model achieves UF1 and UAR of 74.45% and 73.12%. Incorporating the LoRA module improves performance to UF1 75.50% and UAR 76.20%, demonstrating LoRA’s ability to enhance the model’s sensitivity to micro-expressions by adapting the encoder. Adding the SFA module further improves performance to UF1 78.76% and UAR 80.63%, showing SFA’s effectiveness in enhancing spatial feature representation through frequency decomposition and a Mixture of Experts network. The DGAT module boosts performance to UF1 76.40% and UAR 77.43%, confirming DGAT’s contribution to capturing temporal dependencies. Combining LoRA, SFA, and DGAT achieves the best results, with UF1 84.79% and UAR 86.87%, highlighting the significant synergy between all three modules.

### 4.6. The Impact of Loss Function Hyperparameters

To further investigate the impact of hyperparameters $\lambda_{1}$ and $\lambda_{2}$ on model performance within the loss function, we conducted parameter experiments on the three-class task of the CASME II dataset. The loss function is defined as follows:

(30) $$L=L_{\mathrm{CE}}+\lambda_{1}L_{\mathrm{KL}}+\lambda_{2}L_{\mathrm{ST}}$$

where $\lambda_{1}$ and $\lambda_{2}$ control the weights of the KoLeo loss and the spatio-temporal consistency loss, respectively. We evaluated the following parameter combinations:

(31) $$\left\{ \begin{array}{cc} \lambda_{1} & \in \{0.1,\;0.3,\;0.5,\;0.7,\;1.0\}\\ \lambda_{2} & \in \{0.1,\;0.3,\;0.5,\;0.7,\;1.0\} \end{array}\right.$$

**Figure 3:**
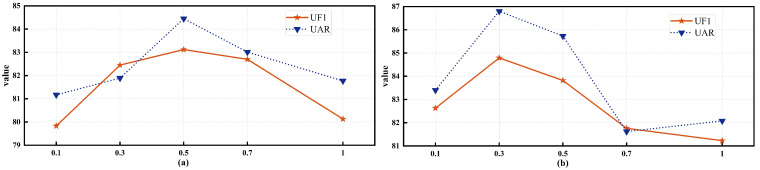
Performance under different loss weight settings. (**a**) Influence of $\lambda_{1}$ on UF1 and UAR with $\lambda_{2}$ fixed at 0.1. (**b**) Influence of $\lambda_{2}$ on UF1 and UAR with $\lambda_{1}$ fixed at 0.5.

Figure 3 illustrates the impact of the loss weighting parameters on model performance. Subfigure (a) shows the variation of UF1 and UAR with different values of $\lambda_{1}$ while fixing $\lambda_{2}=0.1$, and subfigure (b) shows the variation of UF1 and UAR with different values of $\lambda_{2}$ while fixing $\lambda_{1}=0.5$.

The results indicate that when $\lambda_{1}$ is fixed at 0.1, both UF1 and UAR peak at $\lambda_{2}=0.5$. When $\lambda_{2}$ is fixed at 0.5, optimal performance is achieved at $\lambda_{1}=0.3$. Insufficient regularization and consistency constraints occur when $\lambda_{1}$ or $\lambda_{2}$ is too small, leading to degraded performance. Conversely, excessively large values of $\lambda_{1}$ or $\lambda_{2}$ impose overly strong regularization and consistency constraints, which may impair classification capability and reduce performance. These experiments demonstrate that appropriately balancing the loss components is critical for enhancing model performance.

Experimental results on the SAMM, CASME II, and SMIC public datasets validate the superior performance of the proposed MRE model in micro-expression recognition tasks. Parameter sensitivity analysis reveals that the model achieves optimal performance when the loss function parameters are set to $\lambda_{1}=0.5$ and $\lambda_{2}=0.3$. From a broader perspective, the modular design and adaptive feature fusion strategy of the model provides a novel technical paradigm for micro-expression recognition, with potential applicability to other affective computing tasks, such as macro-expression recognition, action unit detection, or cross-modal emotion analysis.

### 4.7. Parameter Efficiency Comparison of LoRA

To assess the parameter-efficient adaptation of the Low-Rank Adaptation (LoRA) mechanism, we compare the fully fine-tuned DinoV2 backbone with the LoRA fine-tuned counterpart under identical SFA and DGAT configurations. Both models are trained on the SAMM dataset using the same optimizer, batch size and learning rate schedule for a fair comparison.

As shown in Table 7, LoRA reduces the number of trainable parameters from 71.2 M to 2.3 M (only 3.2% of full fine-tuning) and lowers the computation from 17.4 G to 15.6 G FLOPs per sample, while decreasing the peak GPU memory from 10.8 GB to 7.2 GB. Despite this reduction in training cost, LoRA slightly improves both UF1 and UAR, demonstrating its ability to maintain DinoV2’s strong representational capacity with much higher efficiency.

Theoretically, LoRA introduces low-rank trainable matrices into the attention projection layers (e.g., $W_{q}$, $W_{v}$), assuming that task-specific parameter updates lie in a low-dimensional subspace. This acts as an implicit regularizer that preserves the geometry of DINOv2’s self-supervised representation space while adapting only the most task-relevant directions. Such low-rank adaptation prevents overfitting on small-scale datasets like SAMM and provides an efficient and stable fine-tuning strategy.

## 5. Conclusions and Discussions

This paper proposes an innovative MER framework to address low-intensity feature extraction, non-uniform temporal dynamics capture, and poor generalization via spatio-temporal interaction modeling. It integrates a LoRA-enhanced DinoV2 encoder, an SFA module for frequency-adaptive spatial enhancement, and a DGAT network with dynamic graph attention and frequency feedback; the framework forms a closed-loop system to overcome core MER bottlenecks. Experimental results on SAMM, CASME II, and SMIC show that the framework outperforms existing methods, achieving state-of-the-art metrics and validating its superiority. Future work will explore cross-dataset transfer learning, multimodal extension, and real-time optimization.

### Interpretability Discussion

In addition to the framework’s performance, interpretability plays a crucial role in real-world applications, particularly in fields like psychology and healthcare. Our model provides transparency by allowing us to trace the model’s decision-making process. The LoRA module enhances the model’s sensitivity to discriminative facial features, while SFA and DGAT contribute by capturing frequency-domain and temporal features, respectively. These features allow us to understand which parts of the face and which time frames are most relevant for recognizing micro-expressions. In the future, we plan to further improve the model’s interpretability by incorporating explicit explanation methods, such as saliency maps and feature importance rankings, to provide even more transparent explanations for the predictions made by the model.

## Figures and Tables

**Table 1:** Class distribution after re-labeling on MER datasets.

Dataset	Setting	Class Distribution (#Samples)	Total
SAMM	3-class	Happiness (26), Surprise (15), Others (118)	159
SAMM	5-class	Happiness (26), Surprise (15), Anger (57), Contempt (9), Others (52)	159
CASME II	3-class	Happiness (33), Surprise (25), Others (189)	247
CASME II	5-class	Happiness (33), Surprise (25), Disgust (60), Repression (27), Others (102)	247
SMIC	3-class	Positive (51), Negative (70), Surprise (43)	164

**Table 2:** MER on SAMM dataset.

Method	Class	UF1 (%)	UAR (%)
AMAN [37]	5	68.10	69.83
SAM-STN [38]	5	71.00	77.33
GRAPH-AU [27]	5	71.23	73.36
MTMNet [39]	5	73.45	74.60
MiMaNet [40]	5	76.60	78.49
MAE [41]	5	80.20	83.10
Ours	5	81.16	85.37
LBT-TOP [8]	3	40.12	41.02
Bi-WOOF [9]	3	62.80	62.18
OFF-ApexNet [24]	3	64.90	63.24
RCN-A [42]	3	75.58	77.83
STANet [12]	3	77.25	78.93
Ours	3	82.93	83.77

**Table 3:** MER on CASME II dataset.

Method	Class	UF1 (%)	UAR (%)
LR-GACNN [28]	5	71.09	75.68
AMAN [37]	5	72.85	74.32
Graph-TCN [44]	5	73.01	79.64
DSTAN [45]	5	74.40	75.03
GEME [46]	5	76.79	77.00
MiMaNet [40]	5	76.83	79.38
SAM-STN [38]	5	79.63	83.07
TSCNN [43]	5	81.93	82.20
Ours	5	83.86	84.77
STSNet [12]	3	80.45	78.68
OFF-ApexNet [24]	3	82.87	85.43
MAE [41]	3	82.94	84.08
Ours	3	84.79	86.87

**Table 4:** MER on SMIC dataset.

Method	Classes	UF1	UAR
DIKD [47]	3	72.14	70.73
TSCNN [43]	3	72.58	72.15
MTMNet [39]	3	75.20	74.98
AMAN [37]	3	76.36	78.26
MiMaNet [40]	3	78.26	77.43
DSTAN [45]	3	78.70	80.03
MAE [41]	3	82.33	81.09
Ours	3	85.72	84.17

**Table 5:** Cross-dataset generalization experiments results.

Method	CASME II		SMIC	
	**UF1**	**UAR**	**UF1**	**UAR**
Ours (w/o DA)	80.15	78.88	82.03	82.67
Ours (w/DA)	82.67	83.45	84.56	83.78
GRAPH-AU	74.67	76.03	79.75	80.34

w/o DA: without domain adaptation; w/DA: with domain adaptation.

**Table 6:** Ablation study results across different datasets.

Configuration	LoRA	SFA	DGAT	UF1	UAR
Baseline (DinoV2)	×	×	×	74.45	73.12
Baseline + LoRA	✓	×	×	75.50	76.20
Baseline + SFA	×	✓	×	78.76	80.63
Baseline + DGAT	×	×	✓	76.40	77.43
Baseline + LoRA + SFA	✓	✓	×	82.02	83.15
Baseline + LoRA + DGAT	✓	×	✓	83.20	84.10
Ours	✓	✓	✓	84.79	86.87

**Table 7:** Comparison between fully fine-tuned DinoV2 and LoRA fine-tuned DinoV2 on the SAMM dataset.

Method	Trainable Params (M)	FLOPs (G)	GPU Memory (GB)	UF1 (%)	UAR (%)
Full Fine-tuning of DinoV2	71.20	17.4	10.8	81.80	82.20
DinoV2 + LoRA Fine-tuning	2.30	15.6	7.2	84.50	83.60

## Data Availability

Publicly available datasets were analyzed in this study.These data can be found here: SAMM: https://helward.mmu.ac.uk/STAFF/m.yap/dataset.php, accessed on 12 January 2024; CASME: https://www.kaggle.com/datasets/muhammadzamancuiisb/casme2-preprocessed-v2/data, accessed on 23 March 2024; SMIC: https://service.tib.eu/ldmservice/dataset/smic, accessed on 8 February 2024.

## Appendix A

### Appendix A.1. Theoretical Analysis

As summarized in Table A1, conventional micro-expression recognition (MER) methods exhibit distinct limitations in integrating frequency and temporal representations within a unified framework. Graph-based models such as Graph-AU and Graph-TCN construct relational or temporal graphs but lack frequency priors, leading to insufficient sensitivity to subtle motion variations. Transformer-based approaches like MAE and μ-BERT provide strong global modeling capacity yet treat spatial and temporal cues as independent, thus overlooking the spectral dynamics that characterize micro-expressions. Similarly, flow-driven methods such as DSTAN explicitly model optical motion but fail to feed frequency information back into temporal reasoning. In contrast, the proposed framework achieves a theoretically consistent fusion of frequency, spatial, and temporal representations through a closed-loop modulation mechanism. Specifically, the Spatial Frequency Adaptive (SFA) module performs a 2D Fourier transform on the DINOv2 feature tensor to obtain spectral components, and a mixture-of-experts gating network computes the relative spectral energy to generate a frequency feedback term $W_{\mathrm{freq}}$ that quantifies motion granularity. This feedback is injected into the Dynamic Graph Attention Temporal (DGAT) module as a modulation coefficient in the attention computation, allowing the temporal graph to adaptively reweight inter-frame dependencies according to spectral importance. Consequently, DGAT becomes a frequency-conditioned temporal reasoning process, in which frequency priors explicitly guide temporal graph attention. The dynamic convolution fusion then aggregates outputs from both branches using sample-dependent convolutional kernels, forming a non-stationary and adaptive spatio–temporal kernel mixture. The overall model is optimized under a composite objective $L=L_{CE}+\lambda_{1}L_{KoLeo}+\lambda_{2}L_{ST}$, which jointly enforces discriminative compactness, isotropic embedding, and temporal coherence. Theoretically, this design introduces a frequency-conditioned prior P(f|X) into the attention manifold, enabling a self-consistent coupling between spectral and temporal domains. Compared with previous methods listed in Table A1, the proposed SFA–DGAT framework thus provides an interpretable, adaptive, and theoretically unified formulation in which spectral cues actively regulate temporal reasoning and feature fusion within a single optimization paradigm.

**Table A1:** Conceptual comparison with representative MER methods.

Method	Input/Backbone	Temporal Modeling	Freq. & Fusion & Objective
Graph-AU [27]	Landmarks/GCN-GAT	Graph Attention	No explicit freq.; static fusion; CE loss
Graph-TCN [44]	Patches/CNN+TCN	Temporal Conv	No explicit freq.; fixed fusion; CE loss
MAE-based [41]	RGB Frames/ViT-MAE	Simple head	No freq.; transfer-learning fine-tune
μ-BERT [17]	RGB Patches/Transformer	Self-Attn	No freq.; self-supervised pretraining
DSTAN [45]	Optical Flow/Dual CNN	2D-3D Conv	Handcrafted motion; no freq → temporal feedback
Ours (SFA + DGAT)	DINOv2 Features/ViT-LoRA	DGAT (GAT + LSTM)	2D-FFT + MoE SFA; $W_{\mathrm{freq}}$ feedback; Dynamic Conv fusion; CE + KoLeo + ST-consistency

### Appendix A.2. Ablation Study on the Temporal Modeling Component

The ablation is conducted on the SMIC dataset under the standard 3-class setting. We compare the full DGAT (GAT → LSTM → pooling) with a variant that removes the LSTM and directly pools the GAT outputs. All other components and training settings follow the main experiments, including the DinoV2 encoder, SFA module, and the LOSO evaluation protocol. Thus, any performance difference can be attributed solely to the inclusion of the LSTM.

**Table A2:** Ablation study on the temporal modeling component in DGAT.

Variant	UF1 (%)	UAR (%)
DGAT w/o LSTM (GAT + Pooling)	83.16	83.53
DGAT (GAT + LSTM + Pooling)	85.72	84.17

As shown in Table A2, removing the LSTM leads to consistent performance drops in both UF1 and UAR on the SMIC dataset. This indicates that, beyond cross-frame graph attention, the LSTM provides additional benefits by explicitly modeling ordered temporal dynamics, which is particularly important for capturing subtle and noisy micro-expression sequences.
